# CircPVT1 attenuates negative regulation of NRAS by let-7 and drives cancer cells towards oncogenicity

**DOI:** 10.1038/s41598-021-88539-3

**Published:** 2021-04-27

**Authors:** Joshua Miguel C. Danac, Reynaldo L. Garcia

**Affiliations:** Disease Molecular Biology and Epigenetics Laboratory, National Institute of Molecular Biology and Biotechnology, University of the Philippines Diliman, 1101 Quezon City, Philippines

**Keywords:** Cancer, Molecular biology, Oncology

## Abstract

Circular RNAs have emerged as functional regulatory molecules whose aberrant expression has been linked to diverse pathophysiological processes. Here, we report that circPVT1 interferes with let-7 binding to NRAS, confirming this axis as one route by which circPVT1 can instigate an oncogenic program in A549 lung cancer cells and HCT116 colorectal cancer cells. CircPVT1 knockdown significantly reduced NRAS levels and attenuated cancer hallmark phenotypes such as proliferation, migration, resistance to apoptosis, cytoskeletal disorganization, and epithelial-mesenchymal transition. The effects of circPVT1 knockdown were at least partially rescued by blocking binding of let-7 to NRAS 3′UTR with a target protector, suggesting that a circPVT1/let-7/NRAS axis exists and acts in cells to reverse NRAS downregulation and favor oncogenicity. While the phenotypic effects of circPVT1 knockdown may be attributable to the global action of circPVT1, the target protection assays resolved the relative contribution of the circPVT1/let-7/NRAS axis specifically.

## Introduction

Circular RNAs (circRNAs) are a class of functional RNAs that have attracted considerable interest in recent years for their roles in physiology and disease. CircRNAs are generated by the head-to-tail back-splicing of exons in parent transcripts^1^. The 3′ end of an exon is joined to the 5′ end of the same or an upstream exon to make a covalently closed circle, generating a back-splice junction unique to the circular isoform and not found in the genomic sequence or the canonical linear transcript^2^. The notion that circRNAs are mere splicing artifacts has since been steadily disproven. They are now known to possess the ability to interact with RNA-binding proteins, transcription complexes, or microRNAs (miRNAs)^3^, serving as sponges, decoys, scaffolds, or recruiters for RNAs and proteins alike, and extending the epigenetic regulation of gene expression^4^.

While exact functions remain to be determined for the majority of circRNAs, many have already been shown to regulate crucial signaling pathways in diseases such as lung cancer, modulating the oncogenic properties of proliferation, invasion, and metastasis^5^. For instance, CDR1as/ciRS-7^6^ and circCCDC66^7^ appear to be oncogenic in colorectal cancer (CRC), while circ-Foxo3^8^ and circMTO1^9^ are tumor suppressive in breast and liver cancer, respectively.

CircPVT1 (hsa_circ_0001821) is a circRNA derived from circularization of the second exon of the long noncoding RNA (lncRNA) PVT1^3,10^. Like its parent gene, circPVT1 has been linked to a variety of cancers—including gastric cancer^11^, head and neck squamous cell carcinoma^12^, osteosarcoma^13^, and particularly non-small cell lung cancer (NSCLC)^14^. In these studies, increased circPVT1 expression was found in both tumors and cancer cell lines. CircPVT1 was found to be upregulated in a circRNA-seq profile of NSCLC, with potential utility as a biomarker^15^. A similar role of circPVT1 in CRC has been reported^16^, though the literature otherwise remains scant on circPVT1 in the context of CRC.

CircPVT1 has been proposed to act as a competitive endogenous RNA (ceRNA) with predicted targets such as miR-125b/E2F2 in gastric cancer^11^ and NSCLC^14^, miR-497 in head and neck cancer^12^, and miR-145 in CRC^16^. However, without direct evidence to unambiguously implicate these miRNAs in mediating circPVT1 function, the exact mechanism remains tentative and unclear.

On the other hand, it has been shown that circPVT1 expression in normal lung fibroblasts inhibits cellular senescence due to its ability to directly bind and sequester the microRNA let-7, subsequently upregulating let-7 target genes^10^. Pulldown assays confirmed the physical binding of circPVT1 and let-7, while RNAi knockdown of circPVT1 showed that it inhibited the senescent phenotype, and provided evidence for its ability to regulate KRAS protein levels in the cell^10^.

Let-7 miRNAs are a well-conserved and widely expressed miRNA family involved in development and disease^17^. Let-7 miRNAs are master tumor suppressors that regulate several oncogenes such as HMGA2^18^ and MYC^19^. Among the chief targets of let-7 are the Ras oncogenes^20^. Following evidence that let-7 downregulated the Ras ortholog in C. elegans, let-60, via binding to its 3ʹ untranslated region (3ʹUTR), it was found that the three major human Ras isoforms (KRAS, NRAS, and HRAS) all contain let-7 complementary sites in their 3ʹ untranslated regions (3ʹUTRs) as well^20^. Luciferase assays using the KRAS and NRAS 3ʹUTRs, and pan-Ras immunostaining of human cell lines transfected with synthetic let-7 mimics or let-7 antisense inhibitors, provided preliminary evidence that let-7 downregulates the human Ras genes^20^.

Accordingly, let-7 is often deficient in cancer, particularly in lung cancer^21–23^ and colorectal cancer^24^, leading to increased expression of proto-oncogenic Ras. Aside from activating Ras mutations, the role of dysregulation and increased Ras expression in tumorigenesis—regardless of mutational status—has also been demonstrated, particularly for NRAS^25–28^.

To sum up, circPVT1 expression is correlated with several malignancies, including NSCLC and CRC. CircPVT1 is known to bind and sequester the tumor suppressor miRNA let-7, and let-7 in turn is known to downregulate NRAS and other oncogenes. However, it has not been shown whether the circPVT1/let-7 axis in particular is similarly important in NSCLC or CRC, nor whether this circRNA-miRNA interaction could tangibly and significantly shift the cellular phenotype towards oncogenicity. We propose that by virtue of inhibiting let-7 activity, circPVT1 upregulates NRAS and thus enhances oncogenic phenotypes in cells. In the present work, we examine this circPVT1/let-7/NRAS axis using A549 lung adenocarcinoma cells, which express significant endogenous levels of circPVT1^10^, as well as HCT116 colorectal carcinoma cells, for which circPVT1 has not been previously investigated or characterized. We investigated the regulatory interactions between circPVT1, let-7, and NRAS in A549 and HCT116 cells and functionalized the impact of circPVT1 expression on cancer hallmark phenotypes. Finally, we performed let-7 target protection assays to attempt to rescue the phenotypes observed upon circPVT1 knockdown and determine whether the let-7/NRAS axis mediates the functional roles of circPVT1.

## Results

### Let-7 and circPVT1 modulate NRAS expression in A549 and HCT116 cells

**Figure 1:**
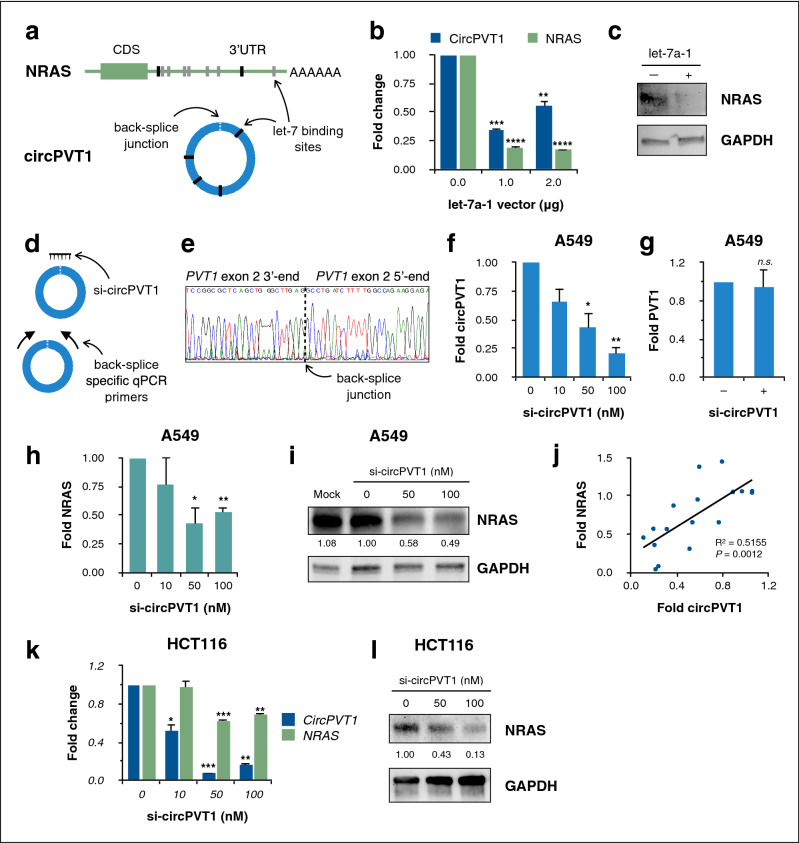
Let-7 and circPVT1 influence NRAS expression in A549 and HCT116 cells. (**a**) Schematic of let-7 binding sites predicted in silico in the NRAS 3′UTR and in circPVT1. CDS, coding region. Black sites are canonical sites predicted in this study, while gray sites are additional noncanonical sites predicted previously^20^. (**b**) RT-qPCR measurements of endogenous circPVT1 and NRAS transcript levels in A549 cells transiently overexpressing let-7a-1. (**c**) Western blot for NRAS protein levels upon let-7a-1 overexpression. (**d**) Schematic of back-splice junction-specific siRNA for circPVT1 knockdown (si-circPVT1, top) and back-splice junction-specific RT-qPCR primers (bottom). (**e**) Sequence verification of the back-splice junction amplified by circPVT1 qPCR primers. (**f**–**h**) RT-qPCR measurements of endogenous (**f**) circPVT1, (**g**) PVT1, and (**h**) NRAS transcript levels in A549 cells upon circPVT1 knockdown. (**i**) Western blot for NRAS protein levels upon circPVT1 knockdown in A549 cells. (**j**) Correlation of fold changes in circPVT1 and NRAS expression as measured by RT-qPCR (n = 17 independent biological replicates). (**k**) RT-qPCR measurements of endogenous circPVT1 and NRAS transcript levels in HCT116 cells upon circPVT1 knockdown. (**l**) Western blots for NRAS protein levels upon circPVT1 knockdown in HCT116 cells. Data shown are fold changes (mean ± S.E.M. from at least three independent experiments) relative to the control set-up. n.s., not significant, *P < 0.05, **P < 0.01, ***P < 0.001, ****P < 0.0001, ANOVA with Dunnett’s post-hoc test. In (**c**) and (**i**), numbers indicate relative GAPDH-normalized densitometric quantity. Full-length blots are shown in Supplementary Figure S1.

We used TargetScan Human v7.2^29^ and microRNA.org^30^ to identify putative let-7 binding sites in the NRAS 3ʹUTR, as well as RNAhybrid^31^ to do the same for the circPVT1 sequence. These tools revealed two major binding sites in the NRAS 3ʹUTR and four in circPVT1 (Fig. 1a; Supplementary Tables S1, S2). The let-7 binding sites predicted in the NRAS 3ʹUTR are attested in the literature^20^.

To validate the in silico predicted interaction of let-7 with both NRAS and circPVT1, a let-7a-1 overexpression construct was transiently transfected into A549 cells. Let-7a is generally the most highly expressed let-7 isoform^17^. Expression of ZsGreen1 fluorescent protein encoded by the pmR-ZsGreen1 miRNA expression vector was used to confirm successful transfection. Overexpression of let-7a-1 was found to reduce NRAS and circPVT1 expression in A549 cells as measured through RT-qPCR (Fig. 1b). Let-7a-1 overexpression also downregulated NRAS at the protein level (Fig. 1c). Previous studies have demonstrated the binding of let-7a to the NRAS 3′UTR^20^ as well as direct binding of let-7 to circPVT1^10^.

Next, we sought to determine whether endogenous circPVT1 in A549 cells had any effect on NRAS expression by means of circPVT1 knockdown. To ensure that knockdown and qPCR amplification were specific to circPVT1 without being confounded by linear PVT1, we used a back-splice junction-specific siRNA (si-circPVT1) targeting the unique sequence not found in linear PVT1, and back-splice junction-specific RT-qPCR primers that only amplify on circPVT1 and are otherwise divergent on a linear template (Fig. 1d). Sequencing of the qPCR product verified the back-splice junction (Fig. 1e).

Successful knockdown of up to about 80 percent of endogenous circPVT1 expression (Fig. 1f) without significantly perturbing PVT1 expression (Fig. 1g) was validated by RT-qPCR. Further, circPVT1 knockdown resulted in a concomitant reduction of NRAS mRNA (Fig. 1h) and protein levels (Fig. 1i) by about half. Regression analysis correlating circPVT1 and NRAS fold changes (Fig. 1j) showed that circPVT1 expression accounted for about half the variation in NRAS (R^2^ = 0.5155, adjusted R^2^ = 0.4832, P = 0.0012). These results imply that circPVT1 promotes and accounts for about half of both NRAS mRNA and protein expression in A549 cells. Similarly, knockdown of endogenous circPVT1 in HCT116 cells resulted in a reduction of NRAS mRNA (Fig. 1k) and protein (Fig. 1l). Thus, circPVT1 upregulates or maintains expression of the oncogene NRAS in both A549 and HCT116 cells.

### CircPVT1 derepresses NRAS by sponging let-7 from its 3′UTR

**Figure 2:**
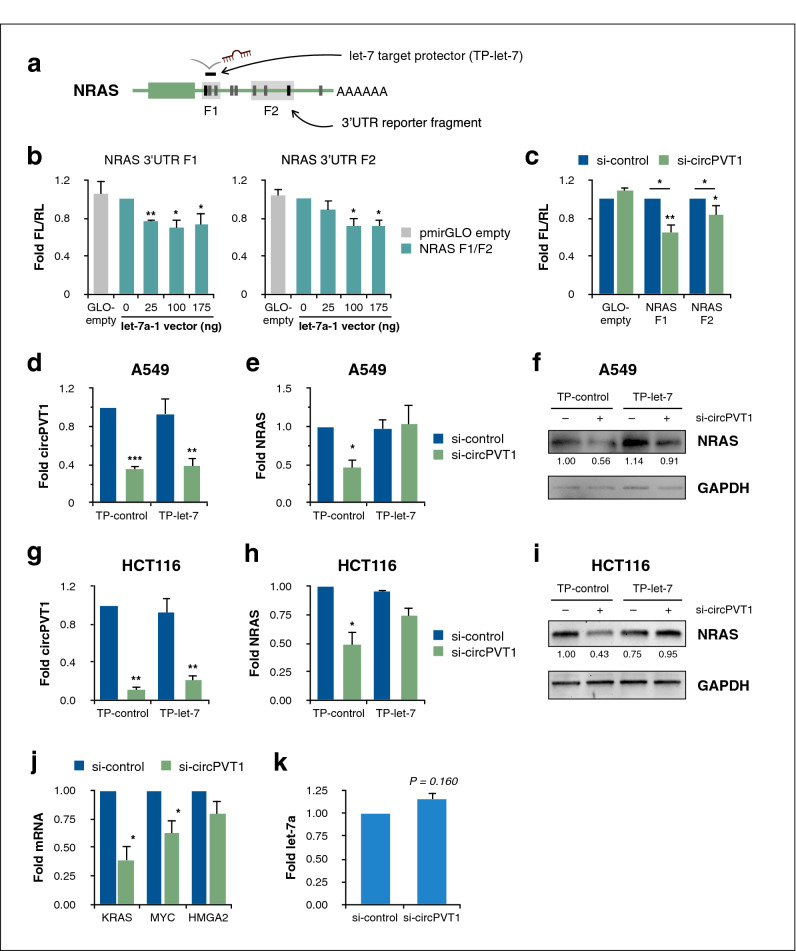
Let-7 binding to the NRAS 3′UTR mediates regulation of NRAS by circPVT1. (**a**) Schematic of the let-7 target protector masking let-7 binding sites in the NRAS 3′UTR. (**b**,**c**) Dual luciferase reporter assays using A549 cells co-transfected with (**b**) NRAS 3′UTR reporter vectors and let-7a-1 expression vector, or (**c**) NRAS 3′UTR reporter vector and either si-control or si-circPVT1. One-sample t-test of fold change against unity with Holm-Šidák correction for multiple comparisons. Additionally, in (**c**), ANOVA with Dunnett’s post-hoc test to compare si-circPVT1 set-ups to vector control. FL/RL, ratio of firefly and Renilla luciferase signals. (**d**,**e**) RT-qPCR measurements of endogenous (**d**) circPVT1 and (**e**) NRAS transcript levels in A549 cells upon co-transfection with either si-control or si-circPVT1 and either negative control target protector (TP-control) or let-7/NRAS 3′UTR target protector (TP-let-7). ANOVA with Dunnett’s post-hoc test. (**f**) Western blot for NRAS protein levels in A549 cells co-transfected as in (**d**,**e**). Numbers indicate relative GAPDH-normalized densitometric quantity. (**g**,**h**) RT-qPCR measurements of endogenous (**g**) circPVT1 and (**h**) NRAS in HCT116 cells, as in (**d**,**e**). (**i**) Western blot for NRAS protein levels in HCT116 cells, as in (**f**). (**j**) RT-qPCR measurements of endogenous transcript levels of let-7 targets KRAS, MYC, and HMGA2 in A549 cells upon circPVT1 knockdown. One-sample t-test of fold change against unity with Holm-Šidák correction for multiple comparisons. (**k**) RT-qPCR measurement of hsa-let-7a-5p in A549 cells upon circPVT1 knockdown. One-sample t-test of fold change against unity. Data are shown as fold changes (mean ± S.E.M. from at least three independent experiments) relative to the control set-up. *P < 0.05, **P < 0.01, ***P < 0.001. Full-length and replicate blots are shown in Supplementary Figure S1.

Having established that circPVT1 and NRAS expression are positively correlated, we next investigated the role of let-7 and its cognate binding sites in the NRAS 3ʹUTR in mediating circPVT1/NRAS crosstalk. Two fragments of the NRAS 3ʹUTR containing putative let-7 binding sites as identified by in silico analyses (Fig. 2a) were cloned into the pmirGLO dual luciferase reporter vector. Transfection of reporter constructs by themselves into A549 cells did not result in any significant repression relative to the empty vector. But upon co-transfection of the reporter constructs with increasing amounts of the let-7a expression vector, a corresponding repression of reporter activity was observed (Fig. 2b). These results imply that let-7 downregulates NRAS via its 3ʹUTR, ostensibly by binding to complementary sites.

If circPVT1 modulates NRAS expression by sponging let-7, then circPVT1 could relieve NRAS repression caused by let-7 binding to complementary sites in the NRAS 3ʹUTR. Indeed, upon circPVT1 knockdown, significant repression was observed for the NRAS 3ʹUTR reporter constructs, but not the empty vector (Fig. 2c). CircPVT1 knockdown thus mimicked the effect of let-7 overexpression without any direct manipulation of let-7 levels otherwise. These findings demonstrate that circPVT1 regulates NRAS through its 3ʹUTR. Furthermore, as the reporter constructs were cloned around the predicted let-7 binding sites, they offer strong evidence that let-7 mediates the circPVT1-NRAS interaction.

To definitively implicate let-7 binding in circPVT1/NRAS regulation, we designed a miScript target protector (a modified ssRNA oligo) to mask the let-7 binding site in the NRAS 3ʹUTR F1—its specificity conferred by complementarity to flanking sequences. The target protector thus abrogates let-7 binding to a specific site in the NRAS 3ʹUTR, without perturbing let-7 interactions with its other targets (Fig. 2a). Upon co-transfection of the target protector with si-circPVT1, the significant decrease in NRAS expression was abolished in both A549 cells (Fig. 2d–f) and HCT116 cells (Fig. 2g–i). Thus, we found that while circPVT1 knockdown reduced NRAS levels, this effect was rescued if let-7 could not bind its target site in the NRAS 3ʹUTR, establishing that a circPVT1/let-7/NRAS axis modulates NRAS expression in cells.

If circPVT1 does inhibit let-7 activity, then we might expect to see effects on other let-7 targets as well. We measured the mRNA levels of other let-7 targets (KRAS, MYC, and HMGA2) upon circPVT1 knockdown, and saw that KRAS and MYC were significantly downregulated (Fig. 2j). This finding lends additional support to the ability of circPVT1 to regulate let-7 and thus modulate the expression of let-7 target transcripts. The absence of an effect on HMGA2 may be explained by either a repression limited to the translational, but not transcriptional, level, as well as the role of context specificity in miRNA-mRNA and other RNA regulatory networks.

While circPVT1 is known to directly bind let-7^10^, the fate of these RNAs upon binding is unknown. In particular, we sought to clarify whether the antagonistic effect of circPVT1 on let-7 was due to a ceRNA mechanism—acting as a let-7 sponge, sequestering the miRNA from downregulating its targets, but without necessarily destabilizing the miRNA and modulating its expression. An alternative possibility is target-directed miRNA degradation (TDMD), a non-ceRNA mechanism of negative regulation of a miRNA, in which binding to a target transcript induces miRNA degradation and turnover^32^. We found that let-7 levels in the cell were not significantly altered upon circPVT1 knockdown (Fig. 2k), suggesting that circPVT1 only influences the availability of let-7, without necessarily modulating its stability and inducing degradation.

### CircPVT1 expression drives a host of cancer hallmark phenotypes

**Figure 3:**
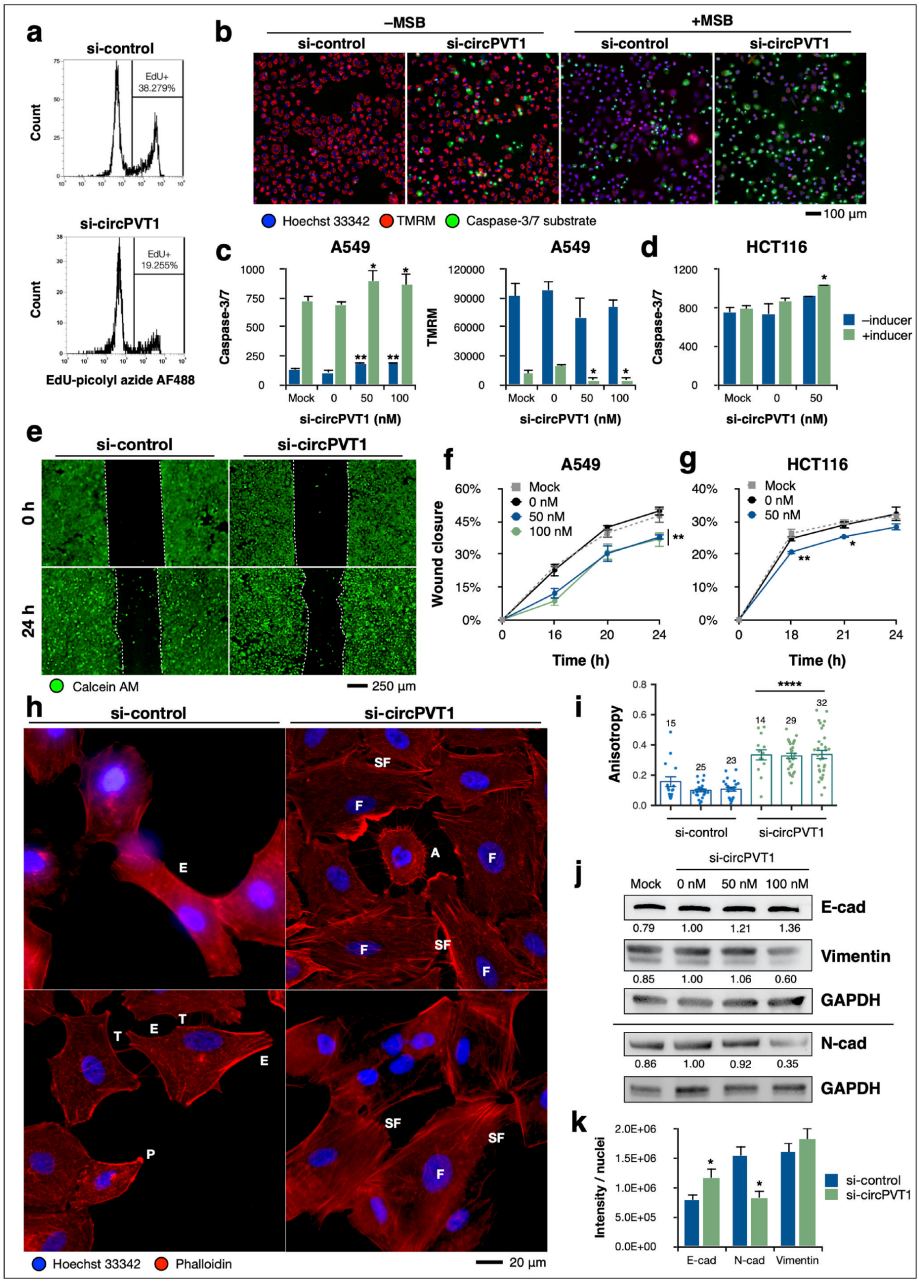
CircPVT1 expression drives a host of cancer hallmark phenotypes. (**a**) Flow cytometric quantitation of EdU-positive A549 cells upon circPVT1 knockdown. (**b**–**d**) Apoptosis assay of (**b**,**c**) A549 cells and (**d**) HCT116 cells upon circPVT1 knockdown, stained with CellEvent caspase-3/7 substrate (green), TMRM for mitochondrial membrane potential (red), and Hoechst 33342 (blue). A549 cells were induced with 100 µM menadione sodium bisulfite (MSB) while HCT116 cells were induced with 5 mM sodium butyrate. Scale bar, 100 µm. (**e**–**g**) Wound healing assay of (**e**,**f**) A549 cells and (**g**) HCT116 cells upon circPVT1 knockdown, stained with vital dye calcein AM (green) for enhanced contrast. (**h**) Phalloidin staining of the F-actin cytoskeleton in A549 cells upon circPVT1 knockdown. P, pseudopodium; E, elongation; T, intercellular tubes; F, flattened and large; SF, stress fibers; A, apoptotic. (**i**) F-actin anisotropy measurements using FibrilTool. Data points are separate individual measurements. Numbers are *n* for each biological replicate. (**j**) Western blot for EMT markers in A549 cells. Numbers indicate relative GAPDH-normalized densitometric quantity. Note that N-cadherin was probed on the same blot as NRAS in Fig. 1i, hence the same GAPDH bands. Full-length blots are shown in Supplementary Figure S1. (**k**) Immunocytochemistry fluorescence quantification of EMT markers in A549 cells. *P < 0.05, two-sample t-test. Data are shown as mean ± S.E.M in a representative of at least three experiments. *P < 0.05, **P < 0.01, ****P < 0.0001, ANOVA with Dunnett’s post-hoc test.

Increased circPVT1 expression has been found in clinical lung cancer cases^14,15^ and lung cancer cell lines^10,14^, as well as in CRC^16^. These observations, along with the known oncogenic properties of Ras, suggest that circPVT1 itself may be oncogenic as well. To investigate this, we knocked down endogenous circPVT1 in A549 and HCT116 cells and subsequently assayed a variety of cancer hallmark phenotypes. Overall, we found that circPVT1 knockdown attenuated the oncogenic characteristics of cancer cells (Fig. 3).

Among the most definitive hallmarks of cancer cells is their increased proliferative capacity^33^. We examined proliferation in A549 cells by quantifying the incorporation of the nucleotide analog 5-ethynyl-2′-deoxyuridine (EdU) into the DNA of actively dividing cells through flow cytometry. CircPVT1 knockdown diminished the proportion of cells that were actively proliferating (Fig. 3a), suggesting that circPVT1 expression promotes increased proliferation.

Another classical phenotype of cancer cells is the ability to evade cell death via apoptosis, even despite triggers that otherwise induce apoptosis in normal cells such as DNA damage or extensive cellular injury^33^. To assess the influence of circPVT1 on resistance to apoptosis, A549 cells under circPVT1 knockdown were treated with menadione bisulfite (MSB), which induces apoptosis through the generation of reactive oxygen species, mitochondrial membrane depolarization, and subsequent intracellular damage^34^. We found that circPVT1 knockdown rendered A549 cells more susceptible to menadione-induced apoptosis (Fig. 3b,c). Notably, even without menadione induction, circPVT1 knockdown resulted in a marked increase in apoptotic cells. The pro-apoptotic effect of circPVT1 knockdown was also recapitulated in HCT116 cells upon induction of apoptosis by treatment with sodium butyrate (Fig. 3d). These results imply that circPVT1 promotes survival and confers resistance to apoptosis in cancer cells.

Increased migration and invasiveness are pro-oncogenic cellular phenotypes involved in metastatic spread^33^. We examined the migratory capacity of A549 cells through a wound healing assay and found that circPVT1 knockdown attenuated the rate of wound healing (Fig. 3e,f). The same effect was also recapitulated in HCT116 cells (Fig. 3g). We can thus infer that circPVT1 expression enhances cellular migratory ability.

Cellular migration typically entails changes in morphology and cytoskeletal organization suited for a more motile phenotype^35^. Accordingly, F-actin fluorescence staining of A549 cells with phalloidin revealed that circPVT1 knockdown resulted in F-actin reorganization (Fig. 3h). Cells under circPVT1 knockdown exhibited increased formation of actin stress fibers and cell-cell adhesion contacts, as well as an overall flattened out and larger morphology. In contrast, cells transfected with control siRNA tended to have diminished stress fibers and more elongated morphology, characteristics that are more associated with motility^35^.

We also quantified F-actin anisotropy using FibrilTool^36^, an ImageJ plug-in used for analyzing fibrillar structures that gives anisotropy values ranging from zero (completely not fibrillar or disorganized) to one (perfectly fibrillar and organized). We found that circPVT1 knockdown increased F-actin anisotropy relative to the control (Fig. 3i), implying increased fibril formation and parallel organization. In motile cancer cells, the F-actin cytoskeleton is highly dysregulated, becoming much more dynamic as it undergoes rapid turnover. As a result, it is disorganized and with fewer focal adhesions to enable cellular motility and invasiveness^35, 37^. We found that these characteristics are associated with circPVT1 expression and attenuated upon circPVT1 knockdown, supporting the role of circPVT1 in enhancing cellular motility and migration.

An integral component of metastasis is the induction of epithelial-mesenchymal transition (EMT)^33^. We found that circPVT1 knockdown resulted in increased expression of the epithelial marker E-cadherin and decreased expression of the mesenchymal markers N-cadherin and vimentin (Fig. 3j), suggesting that circPVT1 drives pro-mesenchymal expression. The same EMT markers were also quantified by immunocytochemistry (Fig. 3k), with circPVT1 knockdown observed to cause reciprocal changes in cadherin expression, as well as leading to a sparser vimentin distribution. Taken together, these results provide evidence for the role of circPVT1 in driving EMT and contributing to the metastatic potential of cancer cells.

### Let-7/NRAS 3′UTR binding facilitates the phenotypic readouts of circPVT1 knockdown

Having examined the phenotypic readouts of circPVT1 expression in A549 and HCT116 cells, we next sought to determine whether the pro-oncogenic role of circPVT1 was due to the circPVT1/let-7/NRAS axis, i.e., whether it was due to circPVT1 relieving let-7 repression of NRAS, upregulating its expression and subsequently driving cancer phenotypes. Since let-7 target protection was able to rescue NRAS expression upon circPVT1 knockdown (Fig. 2), we did further target protection in concert with phenotypic assays to determine whether blocking let-7/NRAS binding would also rescue the effects of circPVT1 knockdown.

**Figure 4:**
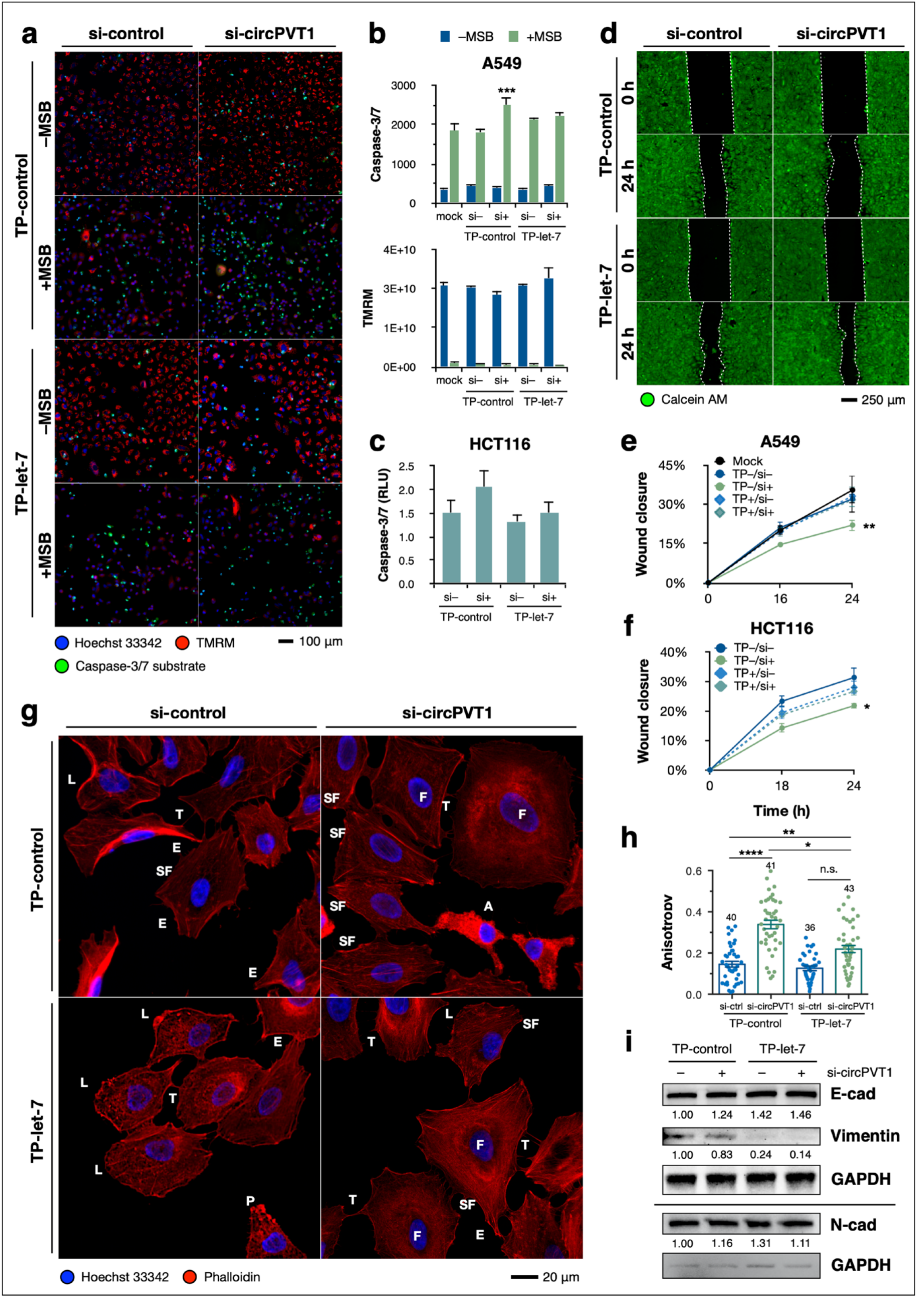
Let-7/NRAS 3′UTR binding facilitates the phenotypic readouts of circPVT1 knockdown. (**a–c**) Apoptosis assay of (**a**,**b**) A549 cells and (**c**) HCT116 cells, co-transfected with si-control or si-circPVT1 and TP-control or TP-let-7, stained with CellEvent caspase-3/7 substrate (green), TMRM for intact mitochondrial membrane potential (red), and Hoechst 33342 (blue). A549 cells were induced with 100 µM menadione sodium bisulfite (MSB) while HCT116 cells were induced with 5 mM sodium butyrate. (**d–f**) Wound healing assay with (**d**,**e**) A549 cells and (**f**) HCT116 cells, co-transfected with si-control or si-circPVT1 and TP-control or TP-let-7, stained with vital dye calcein AM (green) for enhanced contrast. (**g**) Phalloidin staining of the F-actin cytoskeleton in A549 cells, co-transfected with si-control or si-circPVT1 and TP-control or TP-let-7. L, lamellipodium; P, pseudopodium; E, elongation; T, intercellular tubes; F, flattened and large; SF, stress fibers; A, apoptotic. (**h**) F-actin anisotropy measurements using FibrilTool. Data points are separate individual measurements. Numbers are *n* for each biological replicate. (**i**) Western blot for EMT markers in A549 cells, co-transfected with si-control or si-circPVT1 and TP-control or TP-let-7. Numbers indicate relative GAPDH-normalized densitometric quantity. Note that N-cadherin was probed on the same blot as NRAS in Fig. 2, hence the same GAPDH bands. Full-length blots are shown in Supplementary Figure S1. Data are shown as mean ± S.E.M in a representative of at least three independent experiments. *P < 0.05, **P < 0.01, ***P < 0.001, ****P < 0.0001, ANOVA with Dunnett’s post-hoc test.

We found that let-7 target protection of the NRAS 3′UTR partially or completely abolished the sensitization to menadione-induced apoptosis (Fig. 4a,b) and decreased migration (Fig. 4d,e) previously observed upon circPVT1 knockdown in A549 cells. Target protection was also able to rescue the effects of circPVT1 knockdown on apoptosis (Fig. 4c) and migration (Fig. 4f) in HCT116 cells, implying that circPVT1 modulates these hallmarks by relieving the downregulation of NRAS by let-7. Indeed, NRAS is known to enhance survival^38^ and migration^39^ through various downstream effector pathways such as PI3K/Akt and RAF/MEK/ERK. Thus, increased NRAS expression due to circPVT1 may enhance these phenotypes as well in cancer cells.

In terms of F-actin organization, we found that target protection only partially rescued the less motile phenotype observed under circPVT1 knockdown (Fig. 4g), with cells having larger and flatter morphology and stress fiber formation, as well as the persistence of elongations and lamellipodia. Target protection abolished the increase in F-actin anisotropy (Fig. 4h), but stress fiber formation was still apparent under circPVT1 knockdown.

As for EMT, we found that let-7/NRAS target protection alone (without circPVT1 knockdown) resulted in increased E-cadherin and decreased vimentin expression (Fig. 4i). This may be due to target protection leading to a general increased availability of let-7 and resulting in pro-epithelial expression. Overall, our results suggest that the pro-metastatic, motile, mesenchymal phenotype induced by circPVT1 is not solely due to let-7/NRAS, but likely a combination of various other downstream effectors.

## Discussion

The observations that circRNAs are ubiquitous and conserved in eukaryotes, more prevalent than their linear counterparts^40^, temporally and spatially regulated^3^, and independent of their parent genes^41^ strongly imply that circRNAs play significant biological roles—the mechanisms of which have yet to be fully elucidated. Here, we show that circPVT1 is one such circRNA of functional consequence. Through knockdown experiments, we found that circPVT1 tangibly modulates NRAS expression (Fig. 1). Knockdown is the optimal choice for interrogating endogenous circPVT1 function, as genome-level knockout would necessarily disrupt the lncRNA PVT1 at the same locus, and the experimental readouts would be confounded by the loss of PVT1. On the other hand, exogenous overexpression of circPVT1 could lead to artefactual results that do not reflect physiologically relevant interactions, as circRNAs tend to be modestly expressed^42^. We also showed that circPVT1 regulation accounts for about half of NRAS mRNA and protein levels (Fig. 1h–l). Thus, circPVT1 directly affects the level of functional NRAS protein and subsequently drives cancer hallmark phenotypes in cells (Fig. 3).

Let-7/NRAS target protection experiments at least partially reversed the effects of circPVT1 knockdown (Figs. 2 and 4), pointing to let-7 as the crucial link in circPVT1 regulation of NRAS, and linking its downregulation with partially enabling the pro-oncogenic effect of circPVT1. Because miRNAs often have numerous targets within the transcriptome, the choice of a target protector to inhibit miRNA action allows for specific interrogation of the functional sequelae of an individual miRNA-target interaction, as opposed to the use of antisense miRNA inhibitors that could globally upregulate a miRNA’s targets and potentially confound the experimental readout^43^. Thus, we have shown that in the case of circPVT1, its functionality derives, at least partly, from its ability to modulate a particular miRNA-target interaction, regulating an important oncogene and subsequently altering the cellular phenotype.

Crucially, we were able to recapitulate our key findings on the circPVT1/let-7/NRAS axis in both A549 lung cancer cells and HCT116 colorectal cancer cells. In both A549 (Fig. 1h–j) and HCT116 (Fig. 1k,l), knockdown of endogenous circPVT1 reduced NRAS expression by about half. This reduction was dependent on let-7/NRAS 3'UTR binding, and was accordingly rescued by target protection in both cell lines (Fig. 2d–i). As well, the key phenotypes of migration and resistance to apoptosis appear to be modulated by the circPVT1/let-7/NRAS axis in both A549 and HCT116 cells. Taken together, these findings suggest that the oncogenic role of the circPVT1/let-7/NRAS axis may be found across different cancer types, representing a more general mechanism of noncoding RNA-modulated oncogenicity. This could be confirmed by replication of these findings across a wider range of cancer contexts, notwithstanding the particular context dependency of noncoding RNA function.

While our results show that circPVT1 regulates NRAS through let-7, aspects of the interaction between circPVT1 and let-7 remain to be clarified. TDMD has been proposed as an alternative non-ceRNA mechanism through which a transcript can regulate the activity of a miRNA by inducing post-transcriptional modifications such as tailing or trimming that destabilize a miRNA, leading to its decay^44^. Whereas a ceRNA would modulate a miRNA’s availability via competition without necessarily affecting the overall number of miRNA molecules in the cell, TDMD regulation of a miRNA would result in a reduction of actual cellular miRNA levels. We found no significant change in let-7 levels upon circPVT1 knockdown (Fig. 2h), implying that competitive sequestration, rather than miRNA decay, is the prevalent mode of regulation for circPVT1/let-7, altering the miRNA’s availability without affecting its stability. Further experiments will resolve the details of circPVT1/let-7 regulation which may include ceRNA-dependent, ceRNA-independent, or a combination of both mechanisms. Additionally, while our overexpression data suggest that let-7 may reciprocally downregulate circPVT1 (Fig. 1b), whether this is the case at physiological levels of let-7 expression remains to be clarified.

A usual issue when it comes to RNA regulatory networks is whether the involved players are stoichiometrically positioned to effect their proposed roles to any meaningful physiological consequence, especially for ceRNAs^45^. The circular structure of circRNAs makes them more stable not only due to resisting exonuclease attack but also destabilization via decapping or poly(A) deadenylation, the usual mode of miRNA repression in animals^46^. Thus, circRNAs can accumulate to stable levels in the cell, even though lowly transcribed or spliced^42^, and effectively bind and regulate miRNAs while resisting miRNA-induced turnover. We circumvented the question of stoichiometry by employing only knockdown of endogenous circPVT1 and target protection of endogenous NRAS, such that our observed effects are only due to the manipulation of endogenously present factors or interactions.

While this work focused on interrogating the circPVT1/let-7/NRAS axis, the functional role of circPVT1 may not be restricted to this axis alone. Let-7 has several other oncogenic targets, including the other Ras isoforms, and circPVT1 itself may bind and interact with other miRNAs. The phenotypic effects of circPVT1 knockdown (Fig. 3) are attributable to the total action of circPVT1 with its various plausible downstream effectors, while the target protection assays (Fig. 4) resolve which effects are due to the circPVT1/let-7/NRAS axis specifically.

Due to low rates of biogenesis, circRNAs appear to be diluted upon cell division and thus are generally downregulated in proliferative tissues^47^, making the marked upregulation of circPVT1 in NSCLC^14,15^ all the more striking. Our results suggest that said upregulation is due to its pro-oncogenic role, and is likely related to the low let-7 and high Ras expression observed in NSCLC^20^. Transcriptomic profiling of NSCLC and CRC cases may serve to further confirm the correlation between circPVT1 and NRAS expression in lung and colorectal tumors.

NSCLC and CRC are typically associated with mutations in KRAS, not NRAS^48^; in fact, the A549 cell line has a genotype of KRAS G12S and wild-type NRAS^28^, while HCT116 bears the KRAS G13D mutation and wild-type NRAS^49^. Notably, it has been shown that in a mutant KRAS context, wild-type NRAS serves an important cooperative role in enhancing oncogenicity^48^. Our results show a similar role for circPVT1 in two cell lines which carry different activating KRAS mutations. Thus, the impact of circPVT1 action may be more potent through the upregulation of wild-type NRAS that acts cooperatively with mutant KRAS, rather than mutant KRAS itself, which drives oncogenicity through constitutive activation without necessarily being overexpressed—notwithstanding that circPVT1 as well may upregulate KRAS, which also carries let-7 binding sites in its 3ʹUTR^20^.

The cancer cell phenotype is the sum total of several factors, to which circPVT1 may contribute only partially. It remains to be seen what, in turn, is responsible for upregulating circPVT1 expression in cancer, and how the dynamics of the circPVT1/let 7/NRAS axis play out in other contexts. Our findings highlight the importance of the regulatory, in addition to the genetic landscape of cancer, and underscore the role not just of oncogenic mutations but also aberrant regulation. This, in turn, may potentially lay the groundwork for circPVT1 as a molecule of possible diagnostic, prognostic, or therapeutic value.

## Methods

### Cell culture and transient transfection

A549 cells (ATCC CCL-185) were grown in Dulbecco’s Modified Eagle Medium (DMEM) supplemented with 10% fetal bovine serum (FBS) and 2.0 g/l sodium bicarbonate, and incubated at 37 °C, 5% CO_2_. HCT116 cells (ATCC CCL-247) were grown in Roswell Park Memorial Institute (RPMI) 1640 supplemented with 10% fetal bovine serum (FBS) and 2.0 g/l sodium bicarbonate. For routine maintenance, cells were grown to 70–90% confluency before passage. Transfections were carried out at about 18–24 h after seeding in the appropriate culture vessel, with cells at about 80–90% confluency, using the transfection reagent Lipofectamine 2000 (Invitrogen). The required amount of nucleic acid, along with the transfection reagent, was diluted in serum-free DMEM or RPMI, with a total transfection volume of 10% of the maintenance medium volume for the culture vessel.

### Reverse transcription-quantitative PCR (RT-qPCR)

Total RNA was harvested from A549 cells seeded at 150,000 cells/well in 12-well plates, or HCT116 cells seeded at 150,000 cells/well in 24-well plates, about 24 h post-transfection using TRIzol (Thermo Fisher Scientific). cDNA synthesis was then done with 1000 ng RNA as template using M-MLV reverse transcriptase. For qPCR, cDNA was diluted 1:5 or 1:10; each 10-µl reaction had 2 µl template, 5 µl of 2X PowerUp SYBR Green Master Mix (Thermo Fisher Scientific), and gene-specific qPCR primers to a final concentration of 0.4 µM. CircPVT1 was amplified using the back-splice specific divergent primers 5ʹ-CGACTCTTCCTGGTGAAGCATCTGAT-3ʹ (forward) and 5ʹ-TACTTGAACGAAGCTCCATGCAGC-3ʹ (reverse) adopted from a previous study^10^. NRAS was amplified using 5ʹ-CAGTGCCATGAGAGACCAATAC-3ʹ (forward) and 5ʹ-TCTGCTCCCTGTAGAGGTTAAT-3ʹ (reverse). Linear PVT1 was amplified using 5ʹ-CTTCCAGTGGATTTCCTTGC-3ʹ (forward) and 5ʹ-CATCTTGAGGGGCATCTTTT-3ʹ (reverse). Quantification was done using the relative standard curve method and GAPDH was used as the housekeeping control for normalization. Quantification and analysis were done on the Applied Biosystems QuantStudio 3 & 5 Real-Time PCR System, and QuantStudio Design and Analysis Software v1.4.3 (Thermo Fisher Scientific).

For let-7 quantification, microRNA-containing small RNA was extracted from transfected A549 cells using the mirPremier microRNA Isolation Kit (Merck) following the manufacturer’s instructions. MicroRNA polyadenylation and cDNA synthesis was then performed using the MystiCq microRNA cDNA Synthesis Mix. MicroRNA-specific qPCR was performed using the MystiCq Universal PCR primer (Merck, Cat#MIRUP) and the hsa-let-7a-5p specific primer (Merck, Cat#MIRAP00001). RNU6-1 (Merck, Cat#MIRCP00001) and SNORD44 (Merck, Cat#MIRCP00005) were selected as small RNA housekeeping controls.

### Western blotting

Total protein was harvested from A549 cells seeded at 300,000 cells/well in 6-well plates, or HCT116 cells seeded at 200,000 cells/well in 12-well plates, 48 h post-transfection using RIPA lysis buffer supplemented with protease inhibitors. Total lysate protein was quantified through the biconchininic acid (BCA) assay. 30 µg protein was denatured in SDS-PAGE treatment buffer by boiling at 99 °C for 3 min followed by immediate transfer to ice. Samples were run on Any kD Mini-PROTEAN TGX Stain-Free Precast Gels (Bio-Rad) at 30 mA for about 1 h. Proteins were then blotted on a PVDF membrane using the Trans-Blot Turbo Transfer System (Mixed MW protocol: 1.3 A, 25 V, 7 min). Blocking was done using 5% BSA in 1× Tris-buffered saline with 0.1% Tween-20 (TBST). For probing, the following primary antibodies were used: rabbit anti-N-Ras polyclonal antibody (Invitrogen Cat#PA5-34560), rabbit anti-E-cadherin (24E10) monoclonal antibody (Cell Signaling Technology Cat#3195), mouse anti-N-cadherin (13A9) mouse monoclonal antibody (Cell Signaling Technology Cat#14215), rabbit anti-vimentin (D21H3) XP rabbit monoclonal antibody (Cell Signaling Technology Cat#5741), and mouse anti-GAPDH (6C5) monoclonal antibody (Sigma-Aldrich Cat#CB1001). The appropriate species-matched HRP-conjugated secondary antibody was used: goat anti-rabbit IgG (H + L), HRP-conjugated (Invitrogen Cat#31460) or goat anti-mouse IgG (H + L), HRP-conjugated (Invitrogen Cat#31430). For detection, Luminata Classico Western HRP Substrate (Merck) was used, and chemiluminescent imaging was done using ChemiDoc (Bio-Rad) or iBright CL1500 (Invitrogen) imaging systems. For re-probing, membranes were incubated in mild stripping buffer (1.5% glycine, 0.5% SDS, 1% Tween 20, pH 2.2) for 1–2 h. GAPDH was probed for loading control. Total protein was also visualized using stain-free technology.

### Let-7a-1 overexpression

A549 cells were transfected with varying amounts of empty pmR-ZsGreen1 vector or pmR-ZsGreen1-let-7a-1, the total amount of DNA being kept constant. Transfection efficiency was verified to be consistently > 70% by checking the expression of the ZsGreen1 green fluorescent protein. Total RNA or protein was then harvested for RT-qPCR or Western blot.

### CircPVT1 knockdown

CircPVT1 expression was knocked down using a custom back-splice junction specific siRNA (si-circPVT1, Qiagen) with sequence CUUGAGGCCUGAUCUUUUATT following a previous study^10^. The AllStars Negative Control siRNA (Qiagen) was used as the control siRNA (si-control). The siRNAs were transiently transfected into A549 or HCT116 cells to a total concentration of 100 nM (either si-control, si-circPVT1, or a mixture of both). CircPVT1 and PVT1 expression were then measured by RT-qPCR to ensure consistent knockdown (at least > 50%) without significantly affecting PVT1 expression. Following knockdown, downstream RT-qPCR, Western blots, or phenotypic assays were then done.

### In silico analysis of let-7 binding sites in the NRAS 3′UTR and circPVT1 sequences

The NRAS mRNA sequence was obtained from RefSeq (NM_002524.5) and the 3ʹUTR was identified. The online tool TargetScanHuman 7.2^29^ was used to identify putative high-affinity let-7 binding sites according to conserved seed regions, 3ʹ pairing, predicted context scores, and 3ʹUTR prevalence profiles. The identified sites were cross-validated using another online tool, microRNA.org^30^, which also predicts miRNA binding according to sequence and context features. The predicted let-7 binding sites on the NRAS 3ʹUTR were cross-checked against those identified previously in literature^20^. The circPVT1 sequence and genomic reference sequence (hsa_circ_0001821) was obtained from circBase^50^. Putative let-7 binding sites on the circPVT1 sequence were then predicted and analysed using RNAhybrid, an online tool that predicts favorable miRNA-target hybridization sites^31^.

### Cloning of NRAS 3′UTR dual luciferase reporter constructs

Two fragments of the NRAS 3ʹUTR (F1, 282 bp and F2, 933 bp) bearing putative let-7 binding sites were amplified from genomic DNA, extracted from HK-2 human kidney cells (ATCC CRL-2190), using the following primers: F1, GTTTCTCTCGAGTCCCTGGAGGAGAAGTATTCC (forward) and CGTAGGTCTAGATTCACGTTTGCGGTTTGG (reverse); F2, GATTCTCTCGAGGGCCACTTTGTTCCTGTC (forward) and TTCGGGTCTAGATGGTAGCCTTCAGACA GAAC (reverse). The fragments were cloned into the pmirGLO dual luciferase reporter vector (Promega) using the restriction enzymes XhoI and XbaI. Following sequence verification, cell culture-grade constructs for transfection were prepared using the QIAGEN Plasmid Midi Kit.

### Dual luciferase assay

A549 cells were seeded at 10,000 cells/well in 96-well plates and then co-transfected with 25 ng empty or pmirGLO-NRAS 3′UTR and 175 ng empty or pmR-ZsGreen1-let-7a-1 in varying ratios. After verifying high efficiency at 24 h post transfection, the Dual-Luciferase Reporter Assay System (Promega) was done following the manufacturer’s instructions. Briefly, cells were washed with PBS and lysed with 20 µl/well 1× Passive Lysis Buffer for 20 min with shaking at 60 rpm. Debris was spun down at 2500 g for 5 min, and then 5 µl/well of lysate was transferred to an opaque white plate. The FLUOstar Omega multi-mode microplate reader (BMG Labtech) was used for luminescence measurements according to the following protocol: per well, an initial injection of 100 µl Luciferase Assay Reagent, 10 s measurement of luminescence, followed by a second injection of 100 µl Stop & Glo Reagent, and another 10 s measurement, detecting the firefly and Renilla luciferase signals in sequence for each well. For normalization, the firefly luciferase signal from each well was divided by the corresponding Renilla signal. The same assay was also done co-transfecting A549 cells with empty or pmirGLO-NRAS 3′UTR and si-control or si-circPVT1.

### Target protection assay

A custom miScript Target Protector, TP-let-7 (MTP0079424, Qiagen) with the sequence 5ʹ-GAAGTTCTCAGAATAACTACCT CCTCACTTGGCTGTCTGA-3ʹ was designed to mask a let-7 binding site in NRAS 3ʹUTR F1. TP-let-7 or TP-control (Negative Control miScript Target Protector, Qiagen) was co-transfected at 100 nM with 100 nM of either si-control or si-circPVT1. Downstream RT-qPCR, Western blots, and phenotypic assays were then performed as otherwise described.

### Apoptosis assay

Apoptosis was induced in transfected A549 cells grown in black 96-well plates by switching media to DMEM supplemented with 4% FBS and 100 µM menadione sodium bisulfite (MSB, Sigma-Aldrich). HCT116 cells were similarly treated with 5 mM sodium butyrate (Sigma-Aldrich) in RPMI 1640 with 4% FBS. Parallel uninduced set-ups were also maintained. At 16 h post-induction, the media was replenished and supplemented with CellEvent Caspase-3/7 Green Detection Reagent (Invitrogen), Image-iT TMRM reagent mitochondrial membrane potential indicator (Invitrogen), and Hoechst 33342 (Molecular Probes). Cells were incubated at 37 °C, 5% CO_2_, and fluorescence imaging was done using the IN Cell Analyzer 6000 (GE Healthcare) at 20 h post-induction. Image analysis was done with the IN Cell Developer Toolbox software; CellEvent and TMRM fluorescence intensity was quantified and normalized to nuclear count.

### EdU incorporation assay

5-Ethynyl-2ʹ-deoxyuridine (EdU) incorporation in transfected A549 cells was assayed using the Click-iT Plus EdU Flow Cytometry Assay Kit (Molecular Probes). Briefly, transfected A549 cells were incubated in 10 µM EdU at 37 °C, 5% CO_2_ for 2 h and then harvested by trypsinization. 150,000 cells for each set-up was then counted and aliquoted. Click labelling of EdU was then done following the manufacturer’s instructions. Flow cytometry and data analysis was done using the Attune NxT Flow Cytometer (Invitrogen).

### Wound healing assay

Transfected A549 cells or HCT116 cells were grown to full confluency in 96-well plates, and then a white tip was used to create a single scratch in the middle of the cell monolayer in each well. Debris was washed with media and then cells were incubated in the appropriate media with 4% FBS and 10 ng/µl calcein AM (Invitrogen) at 37 °C, 5% CO_2_ for 5 min. The initial wound was photographed using the IN Cell Analyzer 6000 (GE Healthcare). Cells were then incubated at 37 °C, 5% CO_2_. At time points 16–24 h post-scratch, calcein AM was replenished, and cells were photographed at the same fields of view to monitor wound healing. The fluorescence photomicrographs were analyzed using an ImageJ macro to determine the wound area at each time point. Briefly, each image was converted to 8-bit and then thresholded to identify the wound and allow measurement of area. Wound closure was quantified as the change in wound area relative to the initial measurement.

### Phalloidin staining and F-actin anisotropy analysis

A549 cells were fixed with 4% paraformaldehyde in PBS and then permeabilized with 0.1% Triton X-100 in PBS. Blocking was done in 1% BSA in PBS. F-actin was stained using rhodamine phalloidin (Invitrogen) and nuclei were counterstained with Hoechst 33342. Fluorescence photomicrographs were taken with the Olympus IX83 or the IN Cell Analyzer 6000 (GE Healthcare) at several randomized fields of view. Quantitative analysis of F-actin anisotropy was done using the FibrilTool ImageJ plug-in^34^. Anisotropy was measured in several uniform ROIs within visible cells.

### Immunocytochemistry

A549 cells were fixed, permeabilized, and blocked as described above for phalloidin staining. Immunocytochemistry for EMT markers was done separately using the same primary antibodies as for Western blotting. Cells were incubated in 1:500 primary antibody for 1 h, and then washed thrice with 1× PBS, for 5 min each wash. Cells were then incubated in the species-matched secondary antibody: goat anti-mouse IgG (H + L), fluorescein-conjugated (Merck Cat#12-506), goat anti-rabbit IgG (H + L), fluorescein-conjugated (Merck Cat#12-507) or sheep anti-rabbit IgG Cy5 conjugate (Millipore Cat#AP510S) at 1:1000 dilution for 1 h, and then again washed with PBS. Cells were then counterstained with phalloidin and Hoechst 33342 as described above. Fluorescence photomicrographs were taken with the IN Cell Analyzer 6000 (GE Healthcare) at several randomized fields of view. Images were analyzed with the IN Cell Developer Toolbox software, normalizing fluorescence intensity to nuclear count.

### Statistical analysis

Experiments were repeated at least three times and performed with at least three technical replicates in each trial. To compare two means, the unpaired two-tailed t-test was used. To compare three or more means, analysis of variance (ANOVA) with Dunnett’s post hoc test was used to compare to the pertinent control mean. To compare fold changes against the null hypothesis of unity (i.e., no change), the one-sample t-test with Holm-Šidák’s correction for multiple comparisons was used. An overall significance level of *α* = 0*.*05 was used for all statistical tests. Data are reported as mean ± S.E.M. See figure captions for statistical details per experiment.

## Supplementary Information

Supplementary Information.

## Data Availability

All data generated or analysed during the current study are available from the corresponding author on reasonable request.
