# Supplementary material for: CircPVT1 attenuates negative regulation of NRAS by let-7 and drives cancer cells towards oncogenicity
Source: Sci Rep. 2021 Apr 27;11:9021. doi: 10.1038/s41598-021-88539-3 (PMC8079436; doi:10.1038/s41598-021-88539-3)

CircPVT1 attenuates negative regulation of NRAS by let-7 and drives cancer cells towards oncogenicity

Joshua Miguel C. Danac[1] and Reynaldo L. Garcia[1,*]

[1]Disease Molecular Biology and Epigenetics Laboratory, National Institute of Molecular Biology and Biotechnology, University of the Philippines Diliman, Quezon City 1101, Philippines

*Correspondence: reygarcia@mbb.upd.edu.ph

Supplementary Table S1. Prediction of let-7 family microRNA targeting of the NRAS 3'UTR. TargetScan Human v7.2 was used to predict binding sites for let-7 family miRNAs in the NRAS 3'UTR. Context scores are calculated based on features of the target and predicted miRNA-target binding, and weighted according to target abundance. P_{CT} refers to the probability of conserved targeting. Predicted sites were cross-checked with microRNA.org, which gives a mirSVR downregulation score and a PhastCons conservation score, as well as with previously predicted let-7 binding sites in the NRAS 3'UTR.

Position on 3'UTR	miRNA	Site type	Context++ score	Context++ score percentile	Weighted context++ score	Conserved branch length	P_{CT}	mirSVR score	PhastCons score
186-192	hsa-let-7f-5p	7mer-m8	-0.44	97	-0.44	6.955	>0.99	-0.2748	0.6277
186-192	hsa-miR-4500	7mer-m8	-0.47	97	-0.47	6.955	>0.99	—	—
186-192	hsa-let-7g-5p	7mer-m8	-0.43	96	-0.43	6.955	>0.99	-0.2772	0.6277
186-192	hsa-let-7a-5p	7mer-m8	-0.43	96	-0.43	6.955	>0.99	-0.2772	0.6277
186-192	hsa-let-7e-5p	7mer-m8	-0.43	96	-0.43	6.955	>0.99	-0.2772	0.6277
186-192	hsa-miR-4458	7mer-m8	-0.43	96	-0.43	6.955	>0.99	—	—
186-192	hsa-let-7d-5p	7mer-m8	-0.43	96	-0.43	6.955	>0.99	-0.2821	0.6277
186-192	hsa-let-7i-5p	7mer-m8	-0.43	96	-0.43	6.955	>0.99	-0.2748	0.6277
186-192	hsa-let-7c-5p	7mer-m8	-0.43	96	-0.43	6.955	>0.99	-0.2772	0.6277
186-192	hsa-let-7b-5p	7mer-m8	-0.43	96	-0.43	6.955	>0.99	-0.2748	0.6277
186-192	hsa-miR-98-5p	7mer-m8	-0.43	96	-0.43	6.955	>0.99	-0.2772	0.6277
2592-2598	hsa-miR-4500	7mer-A1	-0.30	89	-0.09	4.697	0.87	—	—
2592-2598	hsa-miR-4458	7mer-A1	-0.31	88	-0.09	4.697	0.87	—	—
2592-2598	hsa-let-7f-5p	7mer-A1	-0.26	84	-0.08	4.697	0.87	-0.0288	0.5874
2592-2598	hsa-let-7d-5p	7mer-A1	-0.27	84	-0.08	4.697	0.87	-0.0288	0.5874
2592-2598	hsa-let-7e-5p	7mer-A1	-0.24	82	-0.08	4.697	0.87	-0.0288	0.5874
2592-2598	hsa-let-7c-5p	7mer-A1	-0.24	82	-0.08	4.697	0.87	-0.0288	0.5874
2592-2598	hsa-let-7b-5p	7mer-A1	-0.24	82	-0.08	4.697	0.87	-0.0291	0.5874
2592-2598	hsa-miR-98-5p	7mer-A1	-0.24	82	-0.08	4.697	0.87	-0.0288	0.5874
2592-2598	hsa-let-7i-5p	7mer-A1	-0.24	82	-0.08	4.697	0.87	-0.0291	0.5874
2592-2598	hsa-let-7g-5p	7mer-A1	-0.24	82	-0.08	4.697	0.87	-0.0288	0.5874
2592-2598	hsa-let-7a-5p	7mer-A1	-0.24	82	-0.08	4.697	0.87	-0.0288	0.5874

Supplementary Table S2. Prediction of let-7 family microRNA targeting of circPVT1. RNAhybrid was used to predict binding sites for let-7 family miRNAs in the circPVT1 sequence. Top five binding sites for each let-7 miRNA were predicted according to minimum free energy (mfe) of hybridization. Based on the results, the top putative let-7 binding sites (highlighted gray) were mapped in Figure 1A.

Position on circPVT1	miRNA	MFE (kcal/mol)	Position on circPVT1	miRNA	MFE (kcal/mol)	Position on circPVT1	miRNA	MFE (kcal/mol)
41	hsa-miR-98-5p	-15.6	144	hsa-let-7b-5p	-21.1	264	hsa-let-7d-5p	-21.9
47	hsa-let-7g-5p	-23.6	152	hsa-let-7g-5p	-15.6	264	hsa-let-7a-5p	-19.4
47	hsa-let-7d-5p	-21.5	170	hsa-let-7i-5p	-18.4	264	hsa-let-7b-5p	-19.4
47	hsa-let-7f-5p	-21.2	207	hsa-let-7g-5p	-23.9	264	hsa-let-7c-5p	-19.4
47	hsa-let-7a-5p	-19.6	207	hsa-let-7d-5p	-23.7	268	hsa-let-7i-5p	-18.8
47	hsa-let-7e-5p	-18.7	207	hsa-let-7a-5p	-23.4	294	hsa-let-7g-5p	-17.0
47	hsa-let-7i-5p	-18.7	207	hsa-let-7e-5p	-23.4	300	hsa-let-7f-5p	-18.7
53	hsa-let-7b-5p	-19.9	207	hsa-let-7c-5p	-22.1	300	hsa-let-7a-5p	-18.1
53	hsa-let-7c-5p	-19.9	207	hsa-let-7i-5p	-22.1	300	hsa-let-7c-5p	-18.1
59	hsa-miR-98-5p	-15.1	207	hsa-let-7f-5p	-20.4	300	hsa-let-7d-5p	-18.1
82	hsa-let-7f-5p	-16.0	207	hsa-let-7b-5p	-19.9	314	hsa-let-7e-5p	-19.5
92	hsa-let-7b-5p	-19.7	207	hsa-miR-98-5p	-19.2	333	hsa-let-7f-5p	-15.7
136	hsa-let-7d-5p	-18.5	229	hsa-miR-98-5p	-16.4	341	hsa-let-7e-5p	-18.5
136	hsa-let-7a-5p	-16.8	257	hsa-miR-98-5p	-19.8	375	hsa-let-7e-5p	-20.7
144	hsa-let-7c-5p	-22.1	257	hsa-let-7g-5p	-16.6	389	hsa-let-7i-5p	-19.8

Supplementary Figure S1. Full-length blots. Red boxes indicate cropped portions appearing in the main figures. **a.** Blots in Figure 1c, and a replicate blot from a different trial. **b.** Blots in Figure 1i. **c.** Blots in Figure 1l. **d.** Blots in Figure 2f, and a replicate blot from a different trial. **e.** Blots in Figure 2i. **f.** Blots in Figure 3j, including a replicate blot from a different trial for E-cadherin. **g.** Blots in Figure 4i, including a replicate blot from a different trial for N-cadherin.

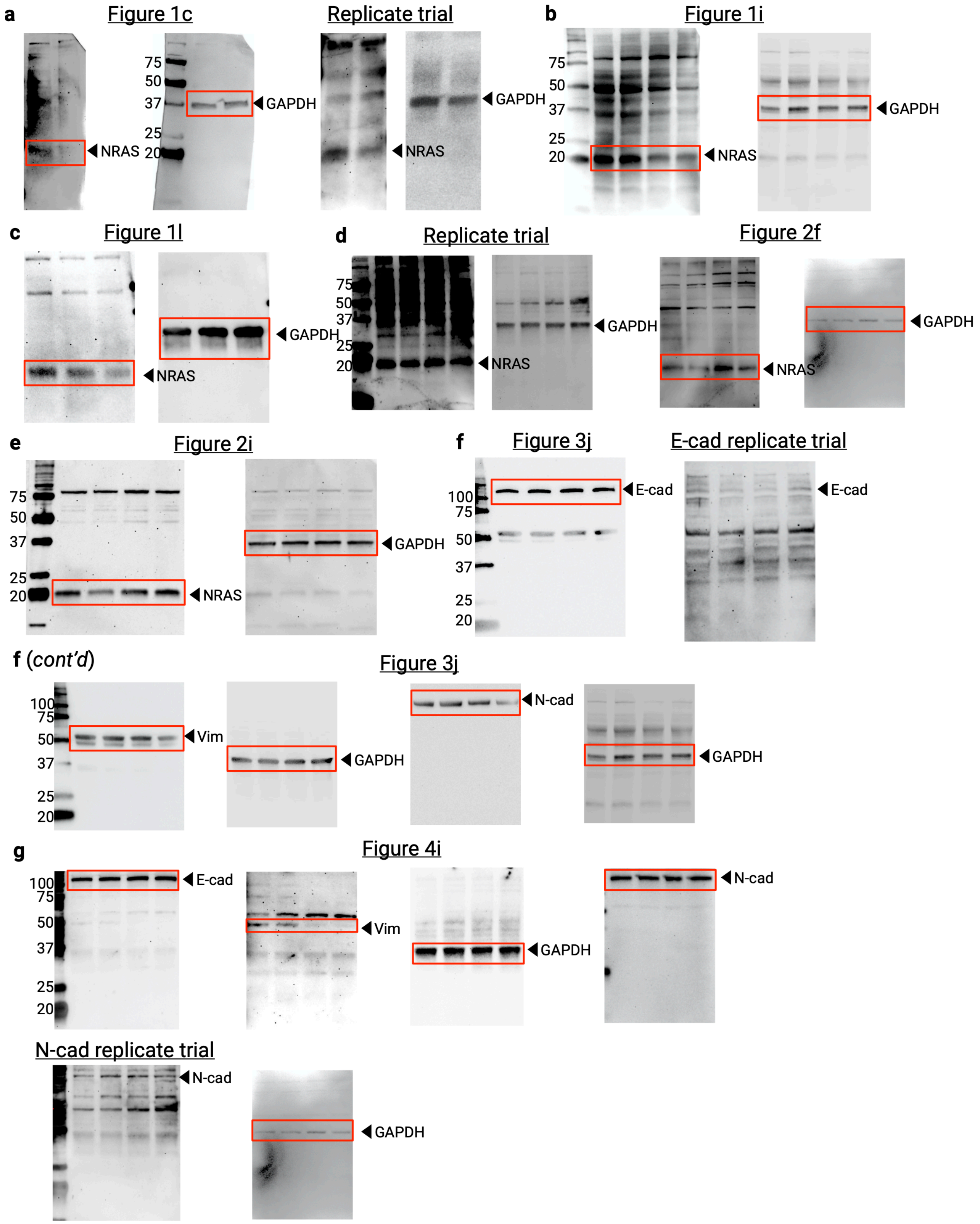

Supplement: Supplementary file 1 — Supplementary Information. [file 41598_2021_88539_MOESM1_ESM.pdf]
